# A condylar imprint of Farabeuf’s knocker and associated cervical variants

**DOI:** 10.1007/s12565-025-00870-1

**Published:** 2025-07-15

**Authors:** Mugurel Constantin Rusu, Răzvan Costin Tudose

**Affiliations:** 1 Division of Anatomy, Department 1, Faculty of Dentistry, “Carol Davila” University of Medicine and Pharmacy, 8 Eroilor Sanitari Blvd., 050474 Bucharest, Romania; 2 Research Department, “Dr. Carol Davila” Central Military Emergency Hospital, 010825 Bucharest, Romania

**Keywords:** Carotid artery, Temporomandibular joint, Mandibular condyle, Hyoid bone, Thyroid cartilage

## Abstract

Different head and neck anatomical structures, such as the temporomandibular joint, carotid arteries and their branches, styloid process, hyoid and thyroid cartilage, are subjected to anatomical variations. A range of novel and rare anatomical variants were found during the anatomical exploration of a DICOM file. The archived angioCT file of a 63-year-old male was examined anatomically. There were no pathological processes or other artefacts to distort the anatomical patterns. The imprint of Farabeuf’s knocker (posterior zygomatic tubercle) on the posterior side of the right mandibular condyle was found. This is an anatomical novelty. Rare neck variations were found: a right-tilted and collapsed hyoid bone over the thyroid cartilage and the agenesis of the left superior horn of this cartilage. Variations of the external carotid arteries’ branches were observed. A right thyrolingual trunk gave off a superior thyroid artery that supplied both thyroid lobes with distinctive right and left branches, the opposite superior thyroid artery being absent. On both sides, the superficial temporal artery coursed over the temporomandibular joints. The right internal carotid artery coiled immediately beneath the skull base. It is recommended that CT scans be carefully explored, as various concomitant anatomical variations, including rare or previously unreported findings, may be encountered.

**Supplementary Information:**

The online version contains supplementary material available at https://doi.org/10.1007/s12565-025-00870-1.

## Introduction

Accurate identification of anatomical landmarks in the head and neck is critical in radiologic interpretation and surgical procedures. However, several potentially important anatomical structures remain underreported or inconsistently described in the literature. The zygomatic root (ZR) is a reliable structure for locating the temporal horn of the lateral ventricle, the temporal tip, and the inferior temporal gyrus (Beckman and Vale 2013). The posterior end of the ZR projects Farabeuf’s knocker (FK, posterior zygomatic tubercle, retromandibular tubercle) (Romero-Reveron and Marin Fermin 2024; Laios et al. 2018) in front of the external auditory canal (Rouviere and Delmas 1985). Thus, the FK arises from the temporal squama, is intraarticular, and completes the mandibular fossa posterosuperiorly. To the best of our knowledge, no studies have been published on the FK since its original description by Louis Hubert Farabeuf (1841–1910).

Similarly, vascular variations in the neck region may occur. The anterior collateral branches of the external carotid artery (ECA) are the superior thyroid (STA), lingual and facial arteries. They may form common trunks, such as the thyrolingual trunk (TLT), linguofacial trunk, or the thyrolinguofacial trunk (Triantafyllou et al. 2024; Lippert and Pabst 1985). These common trunks are a rare occurrence.

This study aims to investigate and characterize novel and rare anatomical variants involving the ZR, FK, hyoid bone (HB), and branches of the ECA, with a particular focus on their spatial relationships and morphological consistency. Given the limited literature on FK, this study aims to expand the current anatomical understanding and establish a clearer foundation for its imaging-based identification and clinical significance.

## Materials and methods

The archived angioCT file of a 63-year-old male was examined anatomically. There were no pathological processes or other artefacts to distort the anatomical patterns. The research followed the principles of the World Medical Association Code of Ethics (Declaration of Helsinki) and was approved by the responsible authorities (approval number 737/01 November 2024, affiliation 2). The imaging used in this study was obtained as part of the patient’s diagnostic workup for suspected cerebral vascular pathology. The patient provided informed consent for using anonymized imaging data for research purposes.

The CT scan was performed as previously, with a 32-slice scanner (Siemens Multislice Perspective Scanner, Forcheim, Germany), with a 0.6 mm collimation and a reconstruction of 0.75 mm thickness, with 50% overlap for a multiplanar maximum intensity projection (Calota et al. 2024). We used the Horos for macOS (Horos Project) software, as in previous studies (Dumitru et al. 2024). Findings were documented on coronal, axial and sagittal slices and three-dimensional volume renderings.

## Results

### Findings at the level of the temporomandibular joint

**Fig. 1:**
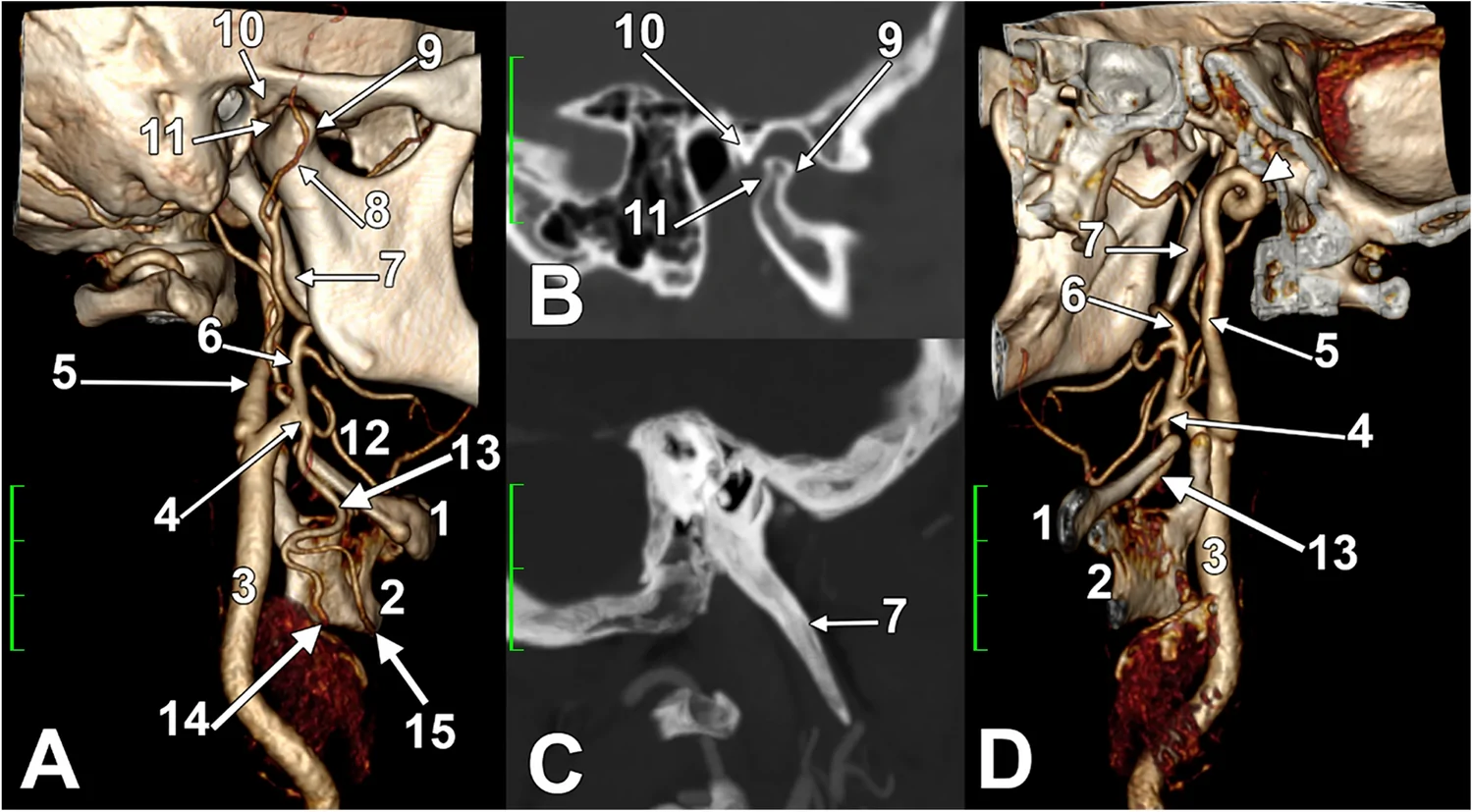
Anatomical variants on the right side. A. Three-dimensional volume rendering, right lateral view. B. Sagittal slice through the right mandibular condyle, right lateral view. C. Sagittal slice through the right styloid process, right lateral view. D. Three-dimensional volume rendering of the right internal carotid artery’s coil (arrowhead), antero-medial view. 1. hyoid body; 2. thyroid cartilage; 3. common carotid artery; 4. thyro-lingual trunk; 5. internal carotid artery; 6. external carotid artery; 7. styloid process; 8. superficial temporal artery; 9. mandibular condyle; 10. Farabeuf’s knocker; 11. imprint of Farabeuf’s knocker; 12. lingual artery; 13. superior thyroid artery; 14. right thyroid branch; 15. left thyroid branch. Scale bar, 1 cm

Triangular FKs were found bilaterally. The distances from the articular tubercles to the FKs were measured. On the left side, it was 8.34 mm, and on the right, it was 10.41 mm. The left was 4.32 mm high, and the right was 3.56 mm high. Although the mandibular fossa was narrower on the left side and the left FK was higher, we found a condylar imprint of the FK of 6.92 mm width and 4.23 mm height on the right side (Fig. 1).

### Findings at the level of the hyoid bone and thyroid cartilage

**Fig. 2:**
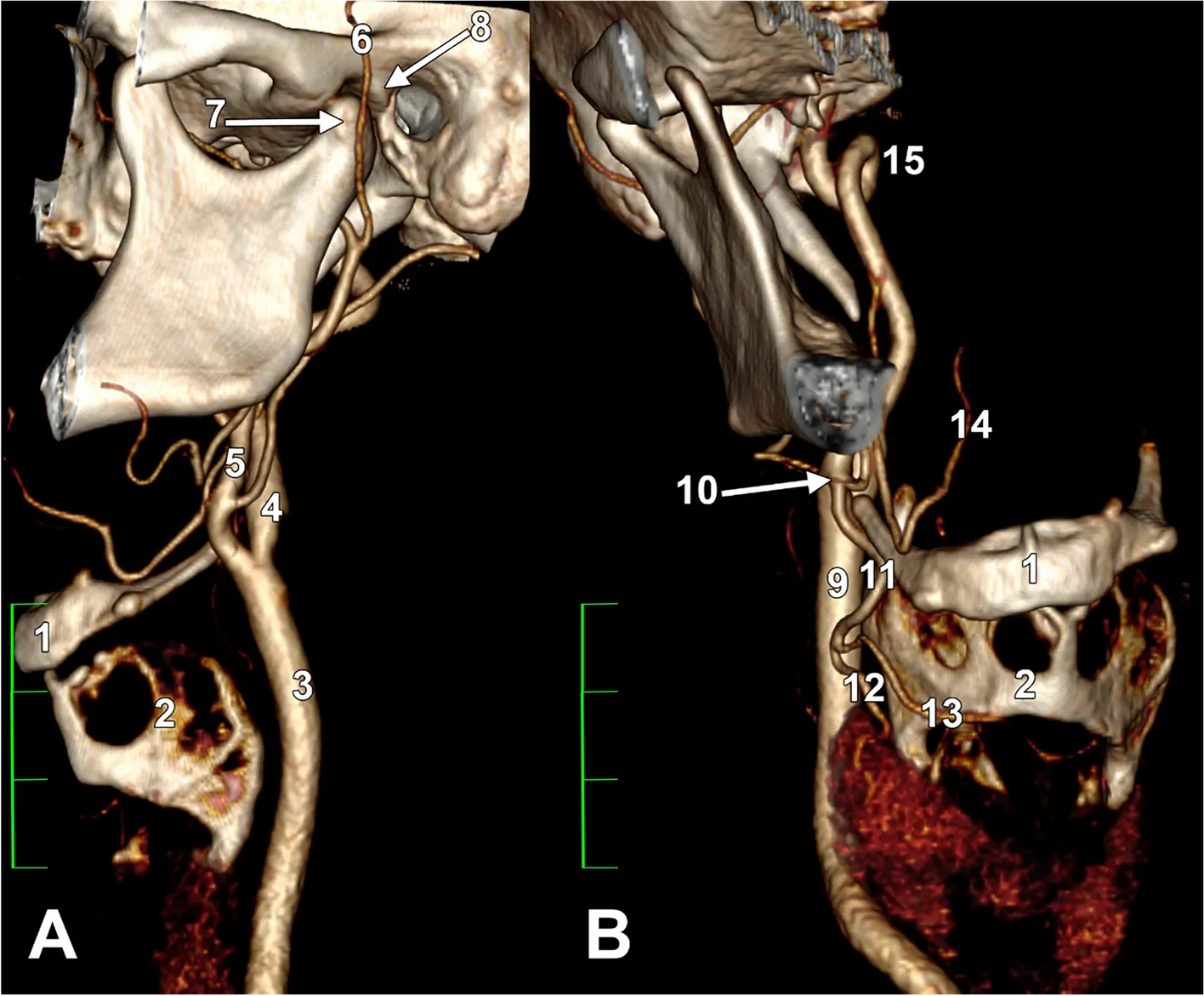
Left and anterior views of the case. Three-dimensional volume renderings. A. Left lateral view. B. Anterior view. 1. hyoid body; 2. thyroid cartilage; 3. left common carotid artery; 4. left internal carotid artery; 5. left external carotid artery; 6. left superficial temporal artery; 7. left mandibular condyle; 8. left Farabeuf’s knocker; 9. right common carotid artery; 10. thyro-lingual trunk; 11. right superior thyroid artery with bilateral distribution; 12. right thyroid branch; 13. left thyroid branch; 14. right lingual artery; 15. coiled right internal carotid artery. Scale bar, 1 cm

A right-tilted and collapsed hyoid bone was also found (Figs. 1 and 2). While the right hyoid tubercle was at the level of the tip of the superior horn of the thyroid cartilage (Fig. 1), the left superior horn of the thyroid cartilage was not discriminated (agenesis of the left superior horn, Fig. 2).

### Arterial variations

Different arterial variations were also found (Supplemental Digital Content). The carotid bifurcations were at the level of the hyoid tubercles. A TLT of 1.92 mm was found on the right side, leaving from the STA just above the right hyoid tubercle (Figs. 1 and 2). It was further divided into the right lingual artery and STA. The left STA was absent. The right STA supplied both thyroid lobes with distinctive right and left branches (Figs. 1 and 2). On both sides, the superficial temporal arteries coursed over the temporomandibular joints.

Long styloid processes were found on both sides. The right was 4.53 cm long, and the left was 3.43 cm long. A coil of the right ICA was detected medially to the right styloid process and antero-inferior to the opening of the hypoglossal canal.

## Discussion

We found different anatomical variants here. The imprint of FKs on the mandibular condyle, the collapsed hyoid, or the agenesis of the superior horn of the thyroid cartilage may not relate to prenatal conditions but to postnatal mechanical conditions. Looping of the ICA may be age-related. Common trunks of the ECA and agenesis of the STA may relate to unbalanced sequences of development in which primordia of target tissues enlarge and elongate their arterial pedicles asymmetrically.

The pooled prevalence of a TLT from the ECA was estimated at 0.61% in a recent meta-analysis (Triantafyllou et al. 2024). It is, therefore, a rare anatomic variation. On the other hand, the absence of the STA is also a rare variation, with a 0.09% pooled prevalence (Triantafyllou et al. 2024). The unilateral absence of the STA may be compensated by the inferior thyroid artery on that side or, as in this study, by the opposite STA. It is, therefore, essential to implement careful surgical management when addressing a STA presenting with bilateral distribution.

The HB may appear tilted or collapsed over the thyroid cartilage of the larynx. Collapsed HB is an atavistic condition that occurs in 3% of cases (Rusu et al. 2024). Agenesis of the superior or inferior horns of the thyroid cartilage is also an infrequent condition and may be complete or incomplete (Hejna et al. 2015). Both the tilted and collapsed HB and the agenesis of the horns of the thyroid cartilage are of interest to forensic pathologists. Moreover, a tilted HB may be part of a broader clinical picture that involves the mandible and the occlusion (Rusu et al. 2024). Analogies between the hyoid, mandible, and malocclusion were previously documented (Rusu et al. 2024). However, a causal relation between the HB malposition, the elongated SP, and the condylar imprint of the FK remains purely speculative.

The posterior slope of the mandibular condyle bears the most significant functional stress (Nishio et al. 2009). Prolonged overloading, such as 10 min of clenching, can lead to cartilage degradation in this region and may contribute to the onset of degenerative joint diseases like osteoarthritis (Nishio et al. 2009). Previous research, however, has identified well-circumscribed posterior slope concavities with a smooth cortical bone lining and normal articular cartilage thickness, both radiographically and microscopically (Hosoki et al. 1996). These findings suggest the absence of cartilage breakdown, supporting the interpretation of such concavities as regressive remodeling—a physiological adaptation of the joint rather than a pathological change (Hosoki et al. 1996). Although the present study relies solely on imaging without histological confirmation, the observed posterior slope concavity closely aligns with these characteristics (Fig. 1B). Therefore, it is reasonable to interpret it as a condylar imprint of the FK, reflecting a functional rather than degenerative, remodelling process, likely caused by a prominent FK.

We also found the superficial temporal arteries coursing over the temporomandibular joints, not posterior. A bilateral laterocondylar superficial temporal artery was found in 13.6% of cases (Rusu et al. 2021). The superficial temporal artery or its branches are used for scalp flaps or intracranial revascularisations (Rusu et al. 2021). Therefore, the anatomical characterisation of this artery should be considered on a case-by-case basis.

Although rare, the coils and kinks of the ICA are not unexpected findings (Paulsen et al. 2000). They may remain clinically silent or lead to cerebrovascular insufficiency or neck symptoms (Paulsen et al. 2000). We found here a coil of the ICA located immediately beneath the floor of the posterior fossa. Such a highly located coil of the internal carotid artery may compress any of the last four cranial nerves and/or the internal jugular vein.

It is recommended to exercise caution when exploring CT scans, as various anatomical variations, including rare or previously unreported findings, may be encountered. Before any exploratory procedures of the head and/or neck, imaging must be completed to avoid iatrogenic injury of potential anatomical variations.

## Supplementary Information

Below is the link to the electronic supplementary material.

Supplementary file1 (MP4 4432 KB)

## Data Availability

The datasets used and analysed during the current study are available from the corresponding author upon reasonable request.

## References

[CR1] Beckman JM, Vale FL (2013) Using the zygomatic root as a reference point in temporal lobe surgery. Acta Neurochir (Wien) 155:2287–2291 24052069 10.1007/s00701-013-1880-0

[CR2] Calota RN, Rusu MC, Vrapciu AD (2024) The external carotid artery and the styloid process. Curr Health Sci J 50:232–236 39380640 10.12865/CHSJ.50.02.08 PMC11459219

[CR3] Dumitru CC, Vrapciu AD, Rusu MC (2024) The diversity of the Linguofacial trunk. Medicina (Kaunas) 60:291 38399578 10.3390/medicina60020291 PMC10890473

[CR4] Hejna P, Janik M, Urbanova P (2015) Agenesis of the superior cornua of the thyroid cartilage: a rare variant of medicolegal importance. Am J Forensic Med Pathol 36:10–12 25376709 10.1097/PAF.0000000000000131

[CR5] Hosoki H, Uemura S, Petersson A, Rohlin M, Akerman S (1996) Concavity of the posterior surface of the temporomandibular condyle: clinical cases and autopsy correlation. Dentomaxillofac Radiol 25:221–227 9161174 10.1259/dmfr.25.5.9161174

[CR6] Laios K, Androutsos G, Zozolou M, Generalis G, Piagkou M, Moschos MM (2018) Louis Hubert Farabeuf (1841–1910). A pioneer of topographical, clinical and surgical anatomy. Ital J Anat Embryol 123:46–50

[CR7] Lippert H, Pabst R (1985) Arterial variations in man: classification and frequency. J.P. Bergmann Verlag, München

[CR8] Nishio C, Tanimoto K, Hirose M, Horiuchi S, Kuroda S, Tanne K et al (2009) Stress analysis in the mandibular condyle during prolonged clenching: a theoretical approach with the finite element method. Proc Inst Mech Eng H 223:739–748 19743639 10.1243/09544119JEIM485

[CR9] Paulsen F, Tillmann B, Christofides C, Richter W, Koebke J (2000) Curving and looping of the internal carotid artery in relation to the pharynx: frequency, embryology and clinical implications. J Anat 197(Pt 3):373–381 11117624 10.1046/j.1469-7580.2000.19730373.x PMC1468139

[CR10] Romero-Reveron R, Marin Fermin T (2024) Louis Farabeuf (1841–1910): Anatomist and inventor of surgical procedures and instruments. J Med Biogr 32:296–300 37221840 10.1177/09677720231177681

[CR11] Rouviere H, Delmas A (1985) Anatomie humaine. Tête et cou, Masson, Paris

[CR12] Rusu MC, Jianu AM, Radoi PM (2021) Anatomic variations of the superficial temporal artery. Surg Radiol Anat 43:445–450 33386932 10.1007/s00276-020-02629-x

[CR13] Rusu MC, Tudose RC, Vrapciu AD, Popescu SA (2024) Lowered hyoid bone overlapping the thyroid cartilage in CT angiograms. Surg Radiol Anat 46:333–339 38315210 10.1007/s00276-024-03300-5

[CR14] Triantafyllou G, Paschopoulos I, Duparc F, Tsakotos G, Tsiouris C, Olewnik L et al (2024) The superior thyroid artery origin pattern: a systematic review with meta-analysis. Surg Radiol Anat 46:1549–1560 39043951 10.1007/s00276-024-03438-2

